# From Blood to Therapy: The Revolutionary Application of Platelets in Cancer-Targeted Drug Delivery

**DOI:** 10.3390/jfb16010015

**Published:** 2025-01-06

**Authors:** Lijuan Xie, Fengxu Gan, Yun Hu, Yibin Zheng, Junshan Lan, Yuting Liu, Xiaofang Zhou, Jianyu Zheng, Xing Zhou, Jie Lou

**Affiliations:** 1 School of Pharmacy and Bioengineering, Chongqing University of Technology, Chongqing 400054, China; shellyxlj@163.com (L.X.); ganfengxu@stu.cqut.edu.cn (F.G.); 15730106266@163.com (Y.H.); zhengyobi@163.com (Y.Z.); 17783568946@163.com (J.L.); liuyuting0404@163.com (Y.L.); deerzhou@stu.cqut.edu.cn (X.Z.); zhengaccordingto@petalmail.com (J.Z.); 2 Chongqing Key Laboratory of Medicinal Chemistry and Molecular Pharmacology, Chongqing University of Technology, Chongqing 400054, China; 3 Yunnan Key Laboratory of Stem Cell and Regenerative Medicine, School of Rehabilitation, Kunming Medical University, Kunming 650500, China

**Keywords:** cancer, platelet, cell membrane, bionic nanosystems, targeted therapy

## Abstract

Biomimetic nanodrug delivery systems based on cell membranes have emerged as a promising approach for targeted cancer therapy due to their biocompatibility and low immunogenicity. Among them, platelet-mediated systems are particularly noteworthy for their innate tumor-homing and cancer cell interaction capabilities. These systems utilize nanoparticles shielded and directed by platelet membrane coatings for efficient drug delivery. This review highlights the role of platelets in cancer therapy, summarizes the advancements in platelet-based drug delivery systems, and discusses their integration with other cancer treatments. Additionally, it addresses the limitations and challenges of platelet-mediated drug delivery, offering insights into future developments in this innovative field.

## 1. Introduction

Cancer, the second leading cause of mortality following cardiovascular diseases, is responsible for approximately one in five deaths globally [1]. Evidence indicates that the origins of human cancer can be traced back approximately one million years. The primary cause of cancer is not an external factor; rather, it arises from the aberrant behavior of the body’s own cells [2]. Cancer is characterized by uncontrolled cell proliferation, resulting in tumor formation. This process may be initiated by genetic damage or somatic mutations [3]. The fundamental characteristics of cancer include self-sufficiency in growth signals, evasion of growth suppressors, resistance to cell death, unlimited replicative potential, induction or access to vasculature, activation of invasion and metastasis, reprogramming of cellular metabolism, and evasion of immune destruction [4]. Despite the growing availability of traditional cancer therapies, millions of patients continue to succumb to cancer-related mortality each year [5]. Conventional treatment methods often fail to completely eradicate cancer and can have adverse effects, such as damaging normal somatic cells and inducing drug resistance within the body [6,7].

Biomimetic nanodrug delivery systems based on cellular components have emerged as a prominent area of research due to their superior biocompatibility and minimal immunogenicity [8,9,10,11]. Among them, platelet-mediated drug delivery systems have garnered significant attention due to their unique biological properties and potential therapeutic benefits. Platelets, anucleate cell fragments derived from megakaryocytes, play a crucial role in hemostasis and wound healing. Beyond their traditional functions, platelets have emerged as promising biopharmaceutical agents owing to their inherent ability to home in on tumor sites, interact with cancer cells, and modulate the tumor microenvironment.

The application of platelets in drug delivery leverages their natural tumor-targeting capabilities and biocompatibility. Platelet integrins αⅡbβ3, α6β1, and P-selectin facilitate the adhesion of platelets to tumor cells. The large volume and surface area of platelets enable efficient drug loading and conjugation. The binding of drugs to platelets can be achieved via covalent or non-covalent interactions. By co-incubating drugs with platelets, the introduction of drugs into platelets occurs through passive diffusion or active uptake mechanisms. Physical or chemical methods may be employed to enhance the permeability of the platelet membrane, thereby facilitating drug entry. Additionally, platelets can deliver drugs by releasing particles or exosomes, which are smaller in size and thus enable broader distribution within the body [12]. Moreover, the low immunogenicity and exceptional tumor-targeting capability of platelets have contributed to their increasing application in nanodrug delivery systems [13].

Platelet-based drug delivery systems typically involve the use of functional nanoparticles encapsulated within bioactive platelet membrane coatings. These systems offer several advantages, including enhanced targeting specificity, reduced immunogenicity, and improved therapeutic efficacy. This paper aims to elucidate the interactions between platelets and cancer, summarize various platelet-based drug delivery strategies, explore the use of platelets in combination with other cancer therapies, and address the limitations and challenges associated with platelet-mediated drug delivery systems [14].

## 2. Physiological Characteristics and Function of Platelets

### 2.1. Physiological Characteristics of Platelet

Platelets are widely distributed in human blood and are anucleate cell fragments derived from bone marrow megakaryocytes, with a diameter of 2–5 μm and an average volume of (6–10) × 10^−9^ L [15]. Platelets, the smallest cells in the circulatory system, typically exhibit a concave, elliptical, or disk-shaped morphology. Their average lifespan is 7–10 days, after which aging platelets are predominantly degraded and metabolized by Kupffer macrophages in the liver and spleen [16,17,18]. The platelet membrane, approximately 6 nm thick, is composed of phospholipids, cholesterol, glycoproteins, lipids, and sugar molecules, which collectively facilitate immune regulation, bioadhesion, and targeted recognition [16,19,20]. For example, integrin αⅡbβ3 is the most abundant glycoprotein on the platelet surface membrane, capable of binding to ligands containing the arginine–glycine–aspartic acid (RGD) sequence. It plays a key role in stabilizing platelet-platelet interactions, adhering to the extracellular matrix, maintaining immune homeostasis in vivo, and facilitating the expression of CD40 ligand on the platelet surface, which stimulates endothelial cells to express adhesion molecules and transcription factors [21].

Platelets contain various organelles, with storage granules such as α-granules, dense granules, and lysosomal granules being the most prominent. These granules store a wide range of protein and non-protein bioactive substances that contribute to platelet physiological functions. CXCL4, the most abundant chemokine in α-granules, plays a crucial role in hemostasis and thrombosis by activating neutrophils, triggering exocytosis, promoting adhesion to the protein matrix or endothelial cells, enhancing monocyte-mediated phagocytosis of invading microorganisms, and facilitating macrophage differentiation, along with other inflammatory pathways [22]. P-selectin, a key soluble protein in α-granules, binds to ligands on endothelial and immune cells, enabling platelets to adhere to endothelial cells at sites of inflammation. This interaction facilitates the recruitment of monocytes, neutrophils, and lymphocytes, thereby initiating inflammatory responses at sites of injury [23].

### 2.2. The Function of Platelets in Pathological Processes

Platelets are involved in various pathological processes within the body. When the vascular endothelium is damaged, circulating resting platelets become activated and play a critical role in hemostasis [24,25]. In instances where vascular integrity is compromised due to vascular injury, chronic inflammation, or rupture of atherosclerotic plaques, platelets can aggregate to form clots at the site of injury [26]. Upon tissue injury, platelets release cytokines and platelet-derived growth factors that promote tissue repair and regeneration while enhancing collagen synthesis [27]. Additionally, platelets are stimulated to produce growth factors that promote vascular growth, such as platelet-derived PDGF receptor-β (PDGFR-β). These receptors bind to endothelial cell-derived PDGF-β, thereby regulating pericyte proliferation and migration during neovascularization [28]. Since the onset of immune thrombocytopenia, platelets have been recognized as playing a crucial regulatory role in both innate and adaptive immune responses. They express various immune-related proteins, including CD47, CD55, CD154, and transforming growth factor-β (TGF-β). Platelets can induce and amplify the inflammatory state by directly interacting with inflammatory cells or by secreting immune mediators through specific glycoproteins on their membrane surface [25,29,30].

In the 1960s, Gasic et al. [31] found that thrombocytopenia induced by intravenous injection of neuraminidase in a mouse model could inhibit cancer metastasis. Recent in vitro and in vivo studies have further demonstrated that platelets can mediate cancer progression by influencing angiogenesis, thrombosis, cancer cell growth, and other mechanisms [32,33].

## 3. Role of Platelets in Tumor Pathogenesis

### 3.1. Changes in Platelets in Tumor Pathogenesis

Platelets are integral components of the tumor microenvironment. Studies have demonstrated that cancer cells can induce platelet production, activation, and aggregation in circulation, while activated platelets can promote tumor growth and metastasis of cancer cells [34]. Tumor-associated humoral factors and cytokines, including granulocyte colony-stimulating factor (G-CSF), granulocyte-macrophage colony-stimulating factor (GM-CSF), interleukin-1 (IL-1), and thrombopoietin (TPO), influence the formation of tumor-associated platelets [35,36,37,38,39]. Inflammatory cytokines, such as tumor necrosis factor-α (TNF-α), interleukin-6 (IL-6), and interleukin-8 (IL-8), as well as platelet agonists like thrombin and ADP, present in the tumor microenvironment, promote platelet autophagy, resulting in platelet activation [34]. Mitrugno et al. [40,41] demonstrated that cancer cells directly induce platelet activation through immunization with the Fcg receptor IIa, promoting the secretion of adenine nucleotide-containing dense granules from platelets. A review of cancer-related clinical data revealed a significant increase in platelet counts among cancer patients [34]. Elevated circulating levels of platelet activation-related markers, such as platelet-specific α-granuloprotein, CD62, CD63, and P-selectin, have been observed in cancer patients [34,42], particularly in the advanced stages of various cancers, such as prostate, breast, and gastric cancers, where levels of β-thromboglobulin (β-TG) are also significantly elevated [43,44]. The frequency of platelet accumulation varies among different types of cancer [34], and platelet count is closely associated with the malignancy of the disease.

### 3.2. The Role of Platelets in Tumor Growth

Studies conducted in a mouse ovarian cancer model have demonstrated that platelets can promote tumor growth, both directly and indirectly, within the tumor microenvironment. Circulating tumor cells (CTCs) release various bioactive factors that activate platelets [44,45]. The α-granules in activated platelets secrete several protumor growth factors, including transforming growth factor-α (TGF-α), transforming growth factor-β (TGF-β), vascular endothelial growth factor (VEGF), and basic fibroblast growth factor (bFGF). These growth factors are released into the bloodstream, facilitating tumor cell proliferation [46,47]. Additionally, platelets promote tumor cell growth through indirect mechanisms that do not rely on growth factors. A highly conserved amino acid residue (EDxxVTPG)3 in the extracellular domain of the transmembrane glycoprotein podoplanin, which is exposed to tumor cells, has been identified as part of the platelet aggregation stimulation (PLAG) domain. The bidirectional signaling between platelet C-type lectin-like receptor 2 (CLEC-2) and podoplanin on tumor cells induces platelet activation, aggregation, and the secretion of bioactive molecules, thereby enhancing tumor cell survival [48]. Blocking the binding of CLEC-2 to podoplanin can inhibit the growth of podoplanin-expressing cancer cells in vivo. Additionally, platelet membranes express CD40L, which promotes tumor growth by interacting with endothelial CD40. Furthermore, platelet activation and aggregation enable tumor cells to evade the immune system and enhance their survival. Nieswandt and Palumbo [49] experimentally demonstrated that tumor cell survival was reduced in rats with thrombocytopenia or a deficiency of proteins essential for platelet activation.

Natural killer (NK) cells play a crucial role in resisting tumor cells and reducing the incidence of metastasis. However, activated platelet-derived factors, such as TGF-β1, can downregulate C-type lectin-like NKG2D receptors, thereby diminishing the antitumor activity of NK cells [50]. Salih [51] demonstrated that platelets reduce the cytotoxic effects of NK cells on tumors by directly transferring MHC Class I molecules to the membranes of tumor cells. Furthermore, platelets can enhance cancer metastasis within the body by releasing substances that increase vascular permeability and facilitate the extravasation of particles. Additionally, they are capable of transporting messenger RNA (mRNA), microRNA (miRNA), and proteins absorbed from their surrounding environment [52,53].

### 3.3. Role of Platelets in Tumor-Associated Thrombosis

Numerous studies have demonstrated that platelets contribute to venous thrombosis, with intracellular platelet-associated proteins such as the platelet target of rapamycin complex 1 (mTORC1) playing a crucial role. mTORC1 promotes platelet activation, increases platelet size, and contributes to the development of venous thrombosis. Inhibition of platelet mTORC1 significantly reduces the incidence of venous thrombosis. Additionally, platelet surface membrane receptors and Von Willebrand Factor (VWF) also play important roles in this process [54]. Cancer patients are at higher risk for venous thromboembolism (VTE) [55]. Cancer cells can activate platelets by secreting platelet activators such as adenosine 5′-diphosphate and by interacting with platelet-specific receptors via their surface membrane proteins. This interaction exposes negatively charged phospholipids, creating a procoagulant surface that induces the production of fibrin and thrombin, thereby promoting thrombus formation. Upon activation, P-selectin, which is stored in platelet α-granules, is expressed on the membrane surface and interacts with P-selectin glycoprotein ligand-1, the primary counter-receptor present on leukocytes, facilitating fibrin formation and thrombosis [55]. Platelets also induce the formation of neutrophil extracellular traps (NETs), which have multiple effects, including the killing of bacteria, activation of platelets and clotting factors, and promotion of cancer-related thrombosis. Studies have demonstrated that the NET marker, citrullinated histone H3, can predict the risk of venous thromboembolism (VTE) in cancer patients [54].

### 3.4. The Role of Platelets in Cancer Angiogenesis

Angiogenesis refers to the formation of new capillaries from the existing vascular system through a predominantly outgrowth mechanism. This process encompasses a series of complex events, including the degradation of the vascular basement membrane, activation of vascular endothelial cells, and the proliferation and formation of migrating new vessels [56]. Angiogenesis plays a pivotal role in both normal physiological processes, such as wound healing, and pathological conditions, including rheumatoid arthritis. It is a key contributor to the formation and maintenance of vascular opacities [57], and is crucial in cancer progression. Neovascularization not only provides the oxygen and nutrients necessary for tumor cell proliferation but also facilitates the transport of proteases and cytokines that enhance tissue invasion and diffusion. Moreover, the incomplete structural characteristics of neovascular networks promote the entry of tumor cells into the bloodstream, thereby contributing to cancer metastasis [58,59].

Numerous studies have demonstrated that components of the hemostatic system play a significant role in promoting angiogenesis. Platelets are implicated in both the early and late stages of this process, where they contribute to the stabilization of newly formed blood vessels [43]. Platelets are capable of storing and expressing over 30 angiogenic regulatory proteins. Von Willebrand Factor (VWF) released by endothelial cells within the tumor vascular system activates platelets, which in turn secrete various angiogenic regulatory factors, including VEGF, epidermal growth factor (EGF), angiopoietin-1, and several anti-angiogenic cytokines. The release of vascular regulatory factors, such as VEGF and endostatin from platelet α-granules, is regulated by protease-activated receptors-1 and -4 (PAR-1 and PAR-4) located on the platelet membrane. Activated platelets facilitate the release of VEGF and endostatin from these α-granules [60]. In theory, platelets can either stimulate or inhibit angiogenesis. However, most studies indicate that the pro-angiogenic effects of platelets predominate over their anti-angiogenic effects. A deficiency in the anti-angiogenic factor thrombospondin-1 (TSP-1) can create an imbalance between pro- and anti-angiogenic factors, consequently accelerating tumor growth and blood vessel formation [43].

## 4. Advances in Platelet-Based Drug Delivery Systems

### 4.1. Mechanisms of Immune Escape and Tumor Targeting in Platelet-Based Drug Delivery Systems

Traditional drug delivery systems, including liposomes, polymer micelles, and nanoparticles, encounter significant challenges such as rapid clearance from the bloodstream and activation of the immune system. These issues hinder their ability to fully satisfy clinical requirements [61]. In contrast, platelet-based drug delivery systems offer a promising solution by circumventing these complex problems [13,62]. Platelet surface CD47 interacts with the inhibitory macrophage receptor signal regulatory protein alpha (SIRRP alpha) to send a signal that inhibits phagocytosis, preventing the platelet from being cleared by immune cells and extending its circulation time in the body [63]. Platelet glycoprotein 1b (GPIb) acts by binding to VWF and adhesion to collagen exposed to damaged blood vessels [64]. Integrin α6β1 on platelets directly interacts with ADAM9 expressed by tumor cells, thereby facilitating efficient lung metastasis. The extracellular matrix protein fibrinogen serves as a bridging molecule between integrin αIIbβ3 on platelets and integrin αvβ3 on tumor cells, mediating the interaction and promoting the formation of platelet–tumor cell aggregates [65,66]. P-selectin on platelets is a vascular cell adhesion molecule that is expressed on activated platelets and rapidly migrates to the membrane. P-selectin promotes the arrest of tumor cells in the vascular endothelium by mutual recognition with the P-selectin glycoprotein ligand-1 (PSGL-1) or CD44 receptor on the surface of tumor cells and contributes to tumor cell metastasis [67,68]. All these provide good biological targeting for platelets to deliver drugs to target sites or target cells. Owing to the ability to target vascular injury sites, strong adhesion capacity, extended circulation time in vivo, and the tumor-homing, circulating tumor cell capture, and targeted migration abilities of activated platelets, they offer an ideal platform for drug encapsulation [69].

### 4.2. Drug Delivery System Based on Platelet Direct Encapsulation

**Figure 1:**
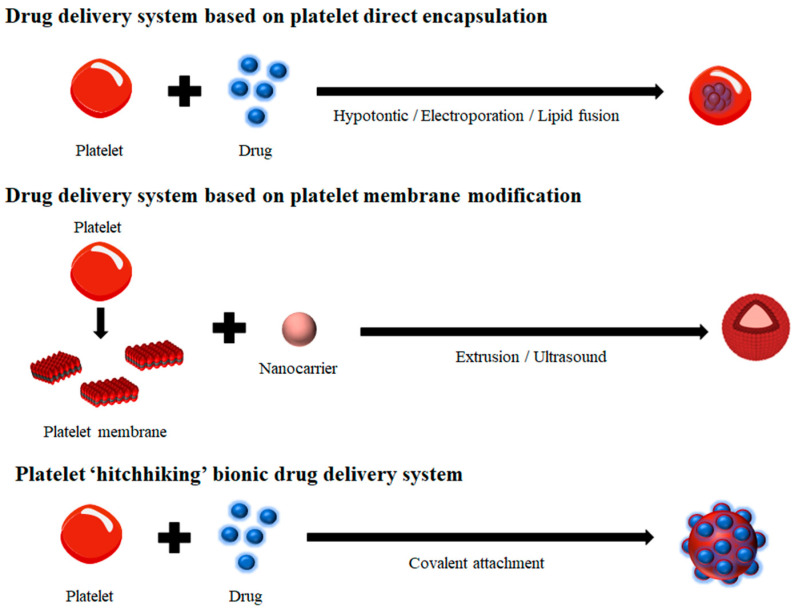
Different platelet-based drug delivery systems.

Encapsulating drugs within intact platelets using methods such as hypotonic treatment, electroporation, and lipid fusion enhances drug stability, delivery efficiency, and therapeutic efficacy (Figure 1). The structure and function of platelets remain unaffected by doxorubicin (DOX), which is encapsulated via the open canalicular system, allowing for high drug-loading capacity and encapsulation efficiency. DOX-loaded platelets facilitate drug accumulation, extending circulation time and improving the therapeutic efficacy of doxorubicin [70]. Photothermal and pH-sensitive chemotherapeutic nanoparticles (PDA@Dox NPs) are encapsulated within drug-loaded platelets, enabling efficient drug loading and controlled release of the therapeutic payload. In this system, platelets facilitate targeted delivery. The photothermal agent IR-820, which possesses fluorescent imaging capabilities, is utilized not only for photothermal therapy but also for imaging guided navigation of platelet carriers. In vivo experiments have demonstrated its excellent therapeutic efficacy and ability to prevent tumor recurrence [71]. DOX attached to nanodiamonds (ND-DOX) was investigated as the model payload drug of platelets. After intravenous injection, a comparison of blood clearance rates revealed that ND-DOX-loaded platelets (Plt@ND-DOX) demonstrated a significantly reduced clearance rate compared to ND-DOX and free DOX. Specifically, free DOX levels plummeted from 0.97 μg/mL to 0.24 μg/mL within 10 h post-administration, whereas Plt@ND-DOX levels decreased more gradually from 1.67 μg/mL to 1.12 μg/mL over 16 h. Notably, Plt@ND-DOX at a dosage of 0.1 mg/kg body weight (bw) exhibited superior antitumor efficacy to DOX at 5 mg/kg bw, without the severe systemic toxicity and associated weight loss observed with DOX treatment [72].

### 4.3. Drug Delivery System Based on Platelet Membrane Modification

The platelet membrane-based nanodrug delivery system addresses the challenges of immune rejection, short half-life, and in vivo toxicity associated with traditional nanocarriers. Studies have shown that the platelet membrane glycoprotein P-selectin interacts with CD44 receptors on the surface of tumor cells, endowing platelets with the capability to target multiple tumor cell lines. Platelet membrane-modified drug delivery systems can be successfully constructed by homogenously coating nanocarriers with platelet membranes obtained through freeze–thaw cycles or osmotic pressure, using extrusion or ultrasound techniques. tPA-Ald-PM-NP, designed for the treatment of multiple myeloma, are composed of bortezomib encapsulated within platelet membranes (PMs) and modified with tissue plasminogen activator (tPA) and alendronate sodium (ALD). ALD targets the bone microenvironment, while P-selectin on the platelet membrane facilitates targeting of myeloma cells, promoting drug accumulation and release at the disease site to more effectively eliminate tumor cells. Pharmacokinetic analyses revealed a reduced clearance rate following platelet membrane coating. Therapeutic efficacy was markedly enhanced in the coated group, as evidenced by a significant prolongation of survival in mice, whereas the bortezomib-only group had a median survival of less than 50 days; median survival in the tPA-Ald-PM-NP group exceeded 80 days for 50% of the mice [73].

Platelet membrane-coated poly (lactic acid)-hydroxyacetic acid copolymer (PLGA) loaded with docetaxel has been employed for the treatment of coronary restenosis, with the platelet membrane specifically targeting type IV collagen exposed at the damaged vessel site. This approach enables precise drug targeting of the lesion and ensures a sustained, slow release of the therapeutic agent for enhanced efficacy [74]. A pH-responsive platelet-based hybrid membrane (pH-HCM) was prepared by co-extruding platelet membranes (CMs) and pH-responsive vesicles (pH-Vs) based on pH-sensitive polymers or lipids, loaded with iron–gallic acid coordination polymer nanodots (FeCNDs). These hybrid membranes, termed pH-HCM@FeCNDs, exhibit potent antitumor activity through hyperthermia and reactive oxygen species generation, owing to their excellent photothermal properties and Fenton-like catalytic performance. pH-HCM@FeCNDs significantly increased intracellular reactive oxygen species levels, induced lipid peroxidation of the cell membrane, and activated Caspase-3, leading to cell death. In a CCK-8 cytotoxicity assay, pH-HCM@FeCNDs demonstrated a strong antitumor effect compared with the control group, while the pH-HCM@FeCNDs + laser group achieved the highest tumor inhibition, with a tumor shrinkage rate of 90.33% in vivo [75].The co-administration of oxymatrine (Om) and astragaloside IV (As) has been shown to augment tumor-infiltrating T lymphocytes (TILs) by inhibiting the activation of cancer-associated fibroblasts (CAFs) and to enhance TIL activity through the improvement of mitochondrial function. A drug-laden nanoplatform, constructed with a magnetic metal–organic framework (MOF) and coated with PM for the delivery of Om and As (PmMN@Om&As), demonstrated a high total drug loading capacity of 33.77 wt% and effective immune evasion. The combination of PmMN@Om&As with α-PD-1 achieved a tumor inhibition rate of 84.15% and extended the survival time of mice [76].

### 4.4. Platelet “Hitchhiking” Bionic Drug Delivery System

The upregulation of platelet membrane surface glycoproteins, such as GPIb and P-selectin, in damaged endothelial or tumor microenvironments enhances platelet adhesion. A platelet “hitchhiking” drug delivery system can be developed by conjugating or modifying drugs onto the surface of platelet membranes through chemical covalent bonding or bioengineering techniques. Covalently bound drugs on the platelet membrane can leverage the targeted binding capabilities of platelets in tumor tissues, circulating tumor cells, injured blood vessels, and other sites to deliver therapeutic agents directly to the lesion. Subsequently, these drugs can activate platelets, prompting the release of drug-containing particles for effective treatment [77]. Antibody-modified hematopoietic stem cell (HSC)-coupled platelets targeting PD-1 were employed in the immunotherapy of postoperative tumor relapse. The results demonstrated that HSC–platelet–aPD-1 (S-P–aPD-1) conjugates significantly enhanced the anti-leukemia immune response, increased the production of active T cells, cytokines, and chemokines, and prolonged the survival time of mice. S-P-APD-1 promotes the targeted accumulation and release of aPD-1 in the bone marrow, thereby unleashing leukemia-specific T cells. This treatment extended the median survival of mice to 80 days, with a survival rate of approximately 87.5%. Furthermore, this cellular conjugate promoted resistance to rechallenge with leukemia cells [78]. Platelets were genetically modified to express surface-bound tumor necrosis factor-related apoptosis-inducing ligand (TRAIL) to inhibit tumor cell metastasis by targeting the interaction between autologous platelets and migrating tumor cells. The results demonstrated that TRAIL-expressing platelets effectively induced the apoptosis of cancer cells in vitro and significantly reduced metastasis in a mouse model of prostate cancer [79]. Glycopolymer micelles conjugated with docetaxel (DTX), which emulate the architecture of P-selectin glycoprotein ligand-1 (PSGL-1), selectively bind to activated platelets, facilitating targeted drug delivery to neoplastic sites. The tumor-targeting efficacy of activated platelets substantially augmented the accumulation of glycopolymer nanoparticles within tumors, with a notable 21.0% drug deposition observed within the initial 0.2 h post-administration [80].

## 5. Application of Platelet-Mediated Drug Delivery Systems in Cancer Therapy

### 5.1. Chemotherapy

**Figure 2:**
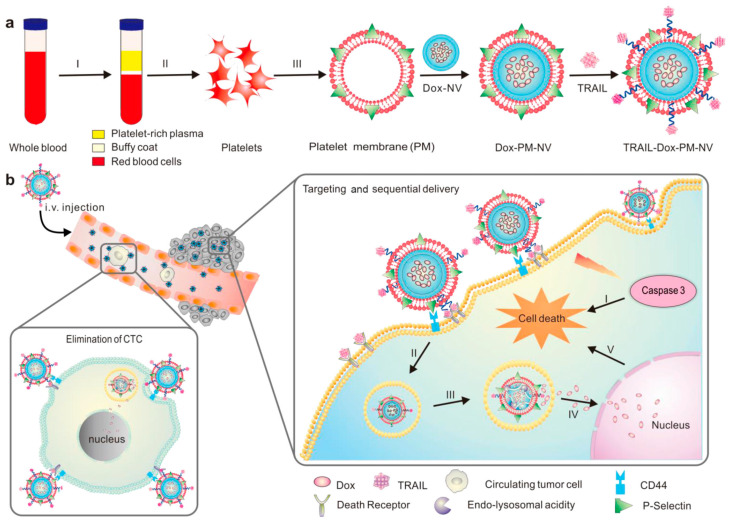
Schematic design of drug-loaded PM-NV for targeting and sequential drug delivery, (**a**),The main components of TRAIL-Dox-PM-NV; (**b**), In vivo elimination of circulating tumor cells (CTCs) and sequential delivery of TRAIL and Dox. reprinted with permission form Ref. [81], Copyright 2025 John Wiley and Sons.

Chemotherapy is a treatment that utilizes chemical agents to inhibit the proliferation, infiltration, and metastasis of cancer cells, ultimately aiming for their eradication. As a systemic therapy, chemotherapy offers a wide range of applications but is often associated with significant toxic side effects. In contrast, platelet-based drug delivery systems exhibit enhanced targeting capabilities, improved therapeutic effects, and reduced toxicity in chemotherapy [81]. A platelet membrane-coated nanomedicine designed for the sequential and site-specific delivery of TRAIL and Dox efficiently targets TRAIL to the cancer cell membrane, thereby activating the extrinsic apoptosis signaling pathway. Additionally, it enhances the accumulation of Dox in the nucleus, facilitating the activation of the intrinsic apoptosis pathway [81] (Figure 2). This drug delivery system not only targets tumor tissue but also eliminates circulating tumor cells, thereby inhibiting distant metastasis. The combination of alendronate (Ald) and bortezomib-loaded nanoparticles coated with tPA-modified platelet membrane creates tPA-Ala-PM-NPs. These nanoparticles aggregate with tumor cells through P-selectin–CD44 binding on the platelet membrane, releasing bortezomib to induce tumor cell death after targeting the myeloma site via Ald. In vitro experiments have demonstrated the excellent targeting capability of both PM-NPs and Ald-PM-NPs, attributed to platelet membrane decoration, as well as increased drug bioavailability due to the sequential targeting strategy for bone and multiple myeloma cells [73]. Chitosan oligosaccharide (CS)-poly (lactic-co-glycolic acid) (PLGA) copolymer loaded with the anti-cancer drug bufoalin was coated with a platelet membrane to form PLTM-CS-pPLGA/Bu nanoparticles (NPs). The PLTM-coated nanoparticles exhibited pH-triggered, sustained drug release over a 48 h period. Confocal microscopy and flow cytometry analyses revealed robust internalization of these nanoparticles by cancer cells. Additionally, the studies validated the active tumor-targeting efficacy of PLTM and the capacity of PLTM-CS-PPLGA/Bu NPs to markedly suppress tumorigenesis without inducing adverse effects on non-target organs [82]. Vascular blocking agents (VDAs) can rapidly obstruct the nutrient supply to tumor cells, leading to their starvation and eventual death. However, angiogenesis-related regrowth enables tumor cells to obtain nutrients from adjacent tissues, thereby diminishing the effectiveness of VDAs and reducing the overall therapeutic efficacy of the treatment [83,84]. Although anti-angiogenic agents (AAs) inhibit angiogenesis and tumor expansion by blocking the interaction between angiogenic factors and their receptors, their therapeutic effects are limited by prolonged treatment durations, the development of drug resistance, and the recurrence of tumors [84,85,86]. Preclinical studies have proved the complementary antitumor effects of AAs and VDAs [87]. Therefore, the combination of VDAs and AAs appears promising for tumor eradication. In this context, platelet membrane-coated mesoporous silica nanoparticles (MSNs) loaded with cobustatin A4 (CA4) and apatinib (Apa) were utilized for targeted tumor therapy. Experimental results indicate that, compared to drugs without encapsulated platelet membranes, MSN@PM-C-A exhibits superior antitumor efficacy. This suggests that platelet membrane coating enhances the antitumor activity of nanoparticles by providing active targeting and self-amplifying accumulation within tumor tissue [87].

### 5.2. Immunotherapy

Tumor immunotherapy controls and eliminates tumor cells by mobilizing the body’s immune system and enhancing antitumor immunity. This approach encompasses various modalities, including antibody drugs, tumor vaccines, adoptive cellular immunotherapy, oncolytic immunotherapy, and biological response modifiers. Platelets can be utilized to target tumor cells, thereby enhancing the effectiveness of immunotherapy. In this study, platelet membrane-camouflaged magnetic nanoparticles were employed for ferroptosis-enhanced cancer immunotherapy. Biomimetic magnetic nanoparticles (Fe_3_O_4_-SAS@PLT) were synthesized by assembling mesoporous magnetic nanoparticles (Fe_3_O_4_) with platelet membranes loaded with sulfasalazine (SAS). Thanks to the self-recognition properties of the platelet membrane, Fe_3_O_4_-SAS@PLT exhibits effective immune evasion and significant tumor metastasis enrichment. Results indicate that Fe_3_O_4_-SAS@PLT not only effectively induces ferroptotic cell death in tumor cells by inhibiting the Xc- transporter pathway of the glutamate–cystine antiporter system but also elicits a robust immune response, thereby enhancing the efficacy of programmed death receptor-1 (PD-1) blockade in vivo [88]. Resiquimod (R848), an immunomodulatory drug, is conjugated to Toll-like receptor (TLR) and can elicit antitumor effects by activating the central transcription factor nuclear factor κB. The targeted delivery of R848 via platelet-coated polylactic acid nanoparticles significantly enhances local immune activation, leading to complete tumor regression in colorectal tumor models while also providing protection against repeated tumor recurrence [89]. The implantation of a hyaluronic acid hydrogel containing CAR-T cells targeting human chondroitin sulfate proteoglycan 4, along with polymer nanoparticles encapsulating the cytokine interleukin-15 and platelets conjugated with the checkpoint inhibitor programmed death-ligand 1 (PD-L1), into the tumor cavity of mice with resected subcutaneous melanoma inhibits local tumor recurrence and suppresses the growth of distant tumors through the abscopal effect. The results indicate that the combination of CAR-T cells and platelets conjugated with anti-PD-L1 (aPD-L1) antibodies covalently linked to the surface of human platelets exhibited enhanced T cell activation and cytokine release in vitro. CAR T-P–aPDL1@gel demonstrated superior antitumor efficacy, with tumor bioluminescence intensity 6.4-fold lower compared to the CAR-T@gel + P-aPDL1 group and over 60-fold lower than other treatment groups. Correspondingly, tumor volume in mice was markedly decreased [90]. The encapsulation of ertatinib (Erda) at the nanoscale within platelet-like theranostics has been utilized to fabricate nano-Erda@PLT, a novel formulation designed to augment the targeted delivery to bladder tumor tissues, thereby eliciting tumor pyrodeath and subsequently bolstering the immune response. In vivo studies focusing on targeted accumulation revealed that a substantial accumulation of the therapeutic agent was observed within subcutaneous tumor tissues following a mere 0.5 h post-treatment interval with nano-Erda@PLT, whereas the distribution of free nanoERDA was predominantly detected in the liver and kidney immediately post-administration. In vitro analyses demonstrated the capacity of nano-Erda@PLT to induce apoptosis in bladder cancer cells. Furthermore, in vivo immunotherapeutic evaluations indicated that the mean overall survival in the nano-Erda@PLT cohort was 37.0 ± 4.38 days, with a tumor inhibition rate reaching a significant 88.31 ± 4.216%. A marked elevation in CD3+CD8+IFN-γ+ cell populations subsequent to nano-Erda@PLT treatment suggests a pronounced activation of cytotoxic T lymphocytes (CTLs) and their consequent antitumor efficacy [91]. Radiation therapy (RT) is a standard cancer treatment, yet its efficacy is compromised by intracellular glutathione (GSH). Platelet membrane biomimetic nanomaterials (PMD) were constructed by loading platelet membrane-encapsulated organic mesoporous silica nanoparticles (MONs) with deoxygen-D-glucose to induce double GSH consumption to enhance tumor radioimmunotherapy. The integration of platelet membrane biomimetic nanomaterials (PMD) with RT resulted in the effective suppression of primary tumor growth and the activation of antitumor immunity. This treatment strategy also led to the regression of distal tumors and a significant prolongation of survival time in mice. Flow cytometry revealed a significant escalation in the proportion of CD8T cells within the PMD+RT cohort, attaining 27.8%, which corresponds to a pronounced enhancement in the tumor infiltration and activation of CD8T cells [92].

### 5.3. Gene Therapy

**Figure 3:**
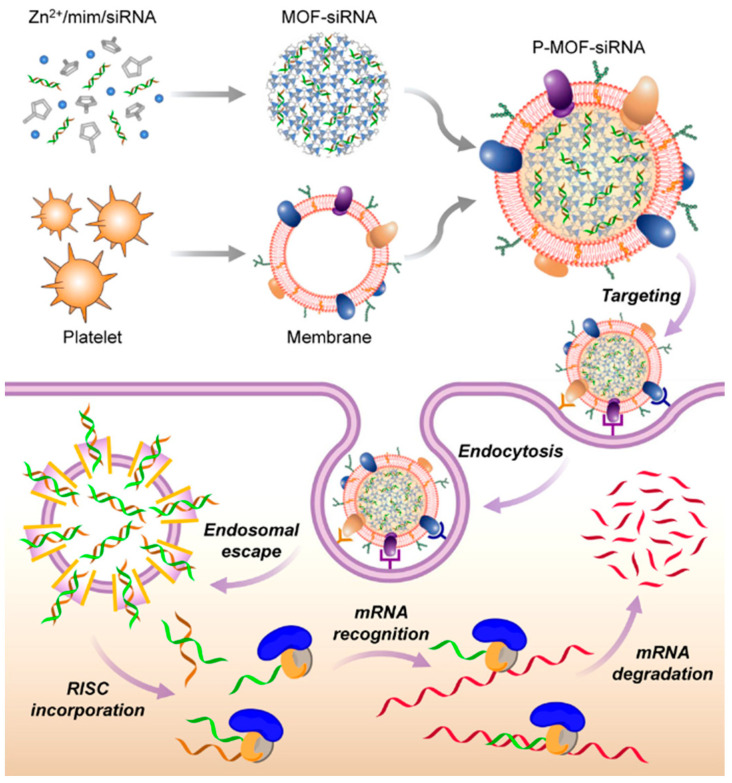
Platelet membrane-coated siRNA-loaded MOFs (P-MOF-siRNA) for gene silencing, reprinted from Ref. [94].

In recent years, tumor treatment has expanded beyond chemical drugs, photothermal agents, and immunomodulatory drugs to include gene delivery approaches. Gene therapy involves the delivery of functional genes into a patient’s body to correct or replace disease-causing genes [93]. Zhuang et al. [94] developed a platelet cell membrane-coated metal–organic MOF delivery platform for the targeted delivery of small interfering RNA (siRNA) in vivo, aimed at inhibiting survivin gene expression in breast cancer and inducing tumor cell death. The platelet membrane enhances the vector’s biocompatibility, reduces its interaction with macrophages, and preserves the integrity of siRNA (Figure 3). Additionally, platelet-like fusogenic liposome-mediated delivery systems can efficiently load and protect miRNA-21 (miR-21) from degradation by RNase. This system facilitates the delivery of miR-21 into the cytoplasm of monocytes/macrophages through membrane fusion, thereby achieving anti-inflammatory reprogramming of inflammatory macrophages [95]. The use of platelet membrane-encapsulated nanocarriers clearly facilitates the effective delivery of nucleic acid drugs, promoting their subsequent clinical translation. Evidence suggests that multi-target elimination of therapeutic miRNAs may be effective in treating malignant tumors. A hybrid membrane vesicle (CPMV) loaded with therapeutic miRNAs for the treatment of triple-negative breast cancer (TNBC) was developed, leveraging the immune system’s clearance ability associated with platelet membrane specific antigen and the active targeting properties of cancer cell membranes. Researchers evaluated the in vivo efficacy of co-delivery of anti-miRNA-10b, anti-miRNA-21, and DOX using CPMV in a TNBC xenograft mouse model. The results indicated that mice administered with a combination of anti-miRNA-CPMV and DOX exhibited a markedly decreased tumor growth rate, with survival times surpassing 60 days in comparison to control cohorts [96].

### 5.4. Photothermal Therapy

**Figure 4:**
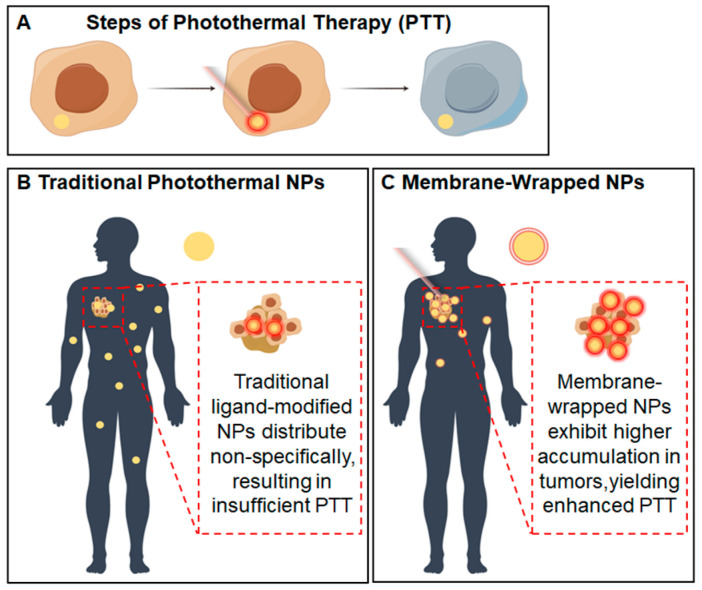
(**A**): Steps of photothermal therapy. (**B**,**C**): The gap between traditional photothermal NPs and membrane-wrapped NPs, adapted with permission form Ref. [97].

Photothermal therapy (PTT) utilizes photothermal agents capable of absorbing light to generate heat, which irreversibly damages cancer cells in the surrounding tumor tissue. Under appropriate conditions, PTT can also induce antitumor immune responses by releasing tumor-associated antigens from ablated tumor cells, thereby preventing or treating metastasis and reducing recurrence. Due to their unique physiological properties, platelet membranes can minimize immune rejection, extend systemic circulation time, and enhance tumor delivery through improved passive accumulation or homotypic targeting, ultimately maximizing the therapeutic efficacy of PTT [97] (Figure 4A–C). Platelet membrane-coated nanoparticles co-loaded with tungsten subcompound (W18O49) and metformin were developed to enhance photodynamic therapy (PDT) and photothermal therapy (PTT). In this system, platelet membranes protect W18O49 from oxidation and facilitate immune evasion while increasing the accumulation of W18O49 at tumor sites through the enhanced permeability and retention (EPR) effect and active adhesion between platelets and cancer cells. Additionally, metformin alleviates tumor hypoxia, ultimately enhancing the therapeutic efficacy of PTT [98].

Platelet membranes were coated onto bismuth selenide (BS) nanoparticles loaded with indocyanine green (ICG) to construct the vector PM@BS-ICG. The platelet membranes help prevent ICG leakage, prolong the circulation time of PM@BS-ICG in the bloodstream, and enhance its accumulation at tumor sites. Upon irradiation with near-infrared light, the platelet membrane is destroyed by the elevated temperature, resulting in the rapid release of ICG from PM@BS-ICG and producing antitumor effects through photothermal action [99]. Platelet membrane-camouflaged mesoporous silica-coated bismuth nanorods (BMSNRs) were utilized to construct the biomimetic material BMSNR@PM. The PM camouflage reduces endocytosis by macrophages in the reticuloendothelial system, thereby enhancing the immune evasion of the BMSNRs. Additionally, this camouflage improves the material’s tumor-targeting capacity, resulting in superior radiotherapeutic efficacy compared to bare BMSNRs. The survival rate of 4T1 cells treated with BMSNR@PM/NIR/IR was significantly reduced to 14.27%, a substantial decrease compared to the rates observed with BMSNR@PM/NIR and BMSNR@PM/IR treatments [100].

## 6. Prospects

Platelets play a crucial role in cancer development by modulating the tumor microenvironment, releasing tumor growth factors, enhancing vascular permeability and extravasation of particles, and attenuating the antitumor effects of circulating NK cells. Due to their close association with cancer, tumor-targeting capabilities, and prolonged circulation time in the body, platelets have emerged as a focal point in anti-cancer therapy. The utilization of platelet-based drug delivery systems has been extensively employed to enhance tumor targeting and therapeutic efficacy. However, their application in chemotherapy, immunotherapy, and gene therapy is primarily limited to targeted carriers and auxiliary drug roles. The impact of various components within platelets on physiology and pathology remains insufficiently explored; thus, further investigation into the role of platelets in anti-cancer therapy is warranted.

## Data Availability

No new data were created or analyzed in this study. Data sharing is not applicable to this article.
